# Case Report: Erdheim-Chester disease presenting as abdominal cocoon syndrome

**DOI:** 10.3389/fimmu.2026.1866684

**Published:** 2026-09-18

**Authors:** Fangfang Liu, Meng Zhang, Fei Chen, Yili Dai, Feng Ding, Mei Ye, Minhu Chen

**Affiliations:** 1 Department of Gastroenterology, Zhongnan Hospital of Wuhan University, Wuhan, Hubei, China; 2 Shenzhen Qianhai Taikang Hospital, Shenzhen, Guangdong, China; 3 Department of Hematopathology, Zhongnan Hospital of Wuhan University, Wuhan, Hubei, China; 4 The Second Clinical College of Wuhan University, Wuhan, Hubei, China; 5 Department of Gastroenterology, The First Affiliated Hospital, Sun Yat-sen University, Guangzhou, Guangdong, China

**Keywords:** abdominal cocoon syndrome, *BRAF^V600E^*, Erdheim-Chester disease, gastrointestinal involvement, vemurafenib

## Abstract

Erdheim-Chester disease (ECD) is a rare histiocytic neoplasm driven by MAPK pathway mutations. Although it frequently involves the bones, heart, and kidneys, initial manifestations predominantly affecting the gastrointestinal tract and mesentery are exceedingly rare, often leading to significant delays in clinical diagnosis. This report describes a 61-year-old female patient who presented with recurrent abdominal pain over several months and was initially diagnosed with a conventional “adhesive bowel obstruction” at an external hospital. However, surgical exploration revealed a classic “abdominal cocoon” presentation and symptoms showed no significant improvement postoperatively. Abdominal CT upon admission revealed diffuse thickening of the peritoneum, omentum, and mesentery with substantial peritoneal and pelvic effusion. Laboratory findings revealed autoimmune serological abnormalities, including positive ANA, anti-SSA, and anti-Ro-52 antibodies, without characteristic clinical manifestations of a defined connective tissue disease. Pathological review of resected specimens revealed extensive foamy histiocytic proliferation in the small intestine and greater omentum, with immunohistochemical findings supporting ECD and molecular confirmation of the *BRAF^V600E^* mutation. Systemic PET-CT further delineated multisystem infiltration, and bone marrow evaluation ruled out the possibility of overlapping myeloid tumors. Following multidisciplinary team (MDT) confirmation, the patient received vemurafenib-based targeted therapy, together with hydroxychloroquine following rheumatologic evaluation for the associated immunological abnormalities, resulting in substantial clinical improvement. This case highlights a rare gastrointestinal manifestation of ECD mimicking a surgical disorder and underscores the importance of integrating imaging, pathology, and molecular testing in patients with atypical intestinal manifestations.

## Introduction

Erdheim-Chester disease (ECD) is a rare histiocytic neoplasm with an incidence of approximately one in ten million. Since its first description in 1930, approximately 1,500 cases have been reported worldwide (1–3). Historically classified as an inflammatory disorder, ECD was reclassified by the WHO in 2016 as an inflammatory myeloid tumor due to its highly frequent mutations in the mitogen-activated protein kinase (MAPK) signaling pathway. The *BRAF^V600E^* mutation is the most common genetic driver, occurring in 57% to 70% of cases, followed by mutations in MAP2K1, NRAS, and KRAS (1).

The clinical manifestations of ECD are highly diverse, with typical features including bilateral symmetrical bone sclerosis of the long bones (80%–95%) and distinctive soft tissue infiltration, such as the imaging-characteristic “coated aorta” (40%–60%) or “hairy kidney” (63%–65%) (4). However, compared to the extensive involvement of other systems, gastrointestinal and mesenteric involvement in ECD is relatively scarce (5). To date, only sporadic literature reports mesenteric ischemia caused by perilesial histiocytic infiltration of the superior mesenteric artery or rare complications such as exudative enteropathy (6, 7).

This report describes a case of an ECD patient presenting with abdominal cocoon syndrome. Through the analysis of this case, we aim to highlight the significant diagnostic pitfalls inherent in such systemic tumors when manifesting with atypical clinical presentations.

## Case presentation

A 61-year-old female was admitted on December 11, 2025, presenting with recurrent abdominal pain for several months and status post enterostomy for one month. Her surgical history included uterine surgery approximately 20 years previously and laparoscopic cholecystectomy one year earlier. She had no previously diagnosed major chronic or autoimmune diseases and was not receiving any long-term medications. Physical examination revealed mild abdominal distension and left lower-quadrant tenderness without rebound tenderness, with multiple scars from previous abdominal surgeries.

In November 2025, she was admitted to an external hospital because of worsening abdominal pain. CT revealed abdominal and pelvic ascites, peritoneal exudative changes, intestinal wall thickening and edema, and right-sided pleural effusion. Cytological examination of pleural and ascitic fluids showed reactive mesothelial and inflammatory cells without evidence of malignancy. Laboratory findings during this hospitalization and the perioperative period are summarized in **Table 1**. Despite conservative treatment including anti-infective therapy, acid suppression, analgesia, antiemetic treatment, intravenous fluid replacement, and nutritional support, her symptoms persisted.

**Table 1:** Selected laboratory findings during the previous hospitalization and perioperative course.

Parameter	Reference range	Preoperative (Nov 3)	POD 4 (Nov 16)	POD 18 (Nov 30)
WBC (×10^9^/L)	3.5–9.5	3.22 ↓	4.87	3.15 ↓
Neutrophils (%)	40–75	79.8 ↑	85.2 ↑	83.5 ↑
Lymphocytes (×10^9^/L)	1.1–3.2	0.48 ↓	0.50 ↓	0.37 ↓
Hemoglobin (g/L)	115–150	108 ↓	89 ↓	82 ↓
Total protein (g/L)	65–85	NA	44.2 ↓	NA
Albumin (g/L)	40–55	NA	20.6 ↓	23.5 ↓
Fibrinogen (g/L)	2.0–4.0	4.76 ↑	NA	NA

POD, postoperative day; WBC, white blood cell count; NA, not available. ↓ indicates a value below the reference range, and ↑ indicates a value above the reference range.

On November 12, 2025, she underwent laparoscopic exploration converted to open laparotomy, partial small-bowel resection, enterostomy, small-bowel repair, and extensive adhesiolysis at the external hospital. Intraoperative findings revealed extensive, plate-like adhesions between intestinal loops and the omentum. A thick fibrous membrane encased the intestinal surfaces, forming a classic “abdominal cocoon” appearance, with particularly dense adhesions in the right paracolic gutter mimicking a mass. Proximal small bowel dilation was also noted. However, her symptoms did not improve significantly postoperatively, prompting further evaluation at our hospital.

Upon admission, abdominal and pelvic CT revealed postoperative changes in the middle and lower abdomen, diffuse thickening of the peritoneum, omentum, and mesentery with intestinal adhesions, massive abdominal and pelvic ascites, and patchy sclerotic lesions in the L1–2 vertebral bodies (**Figure 1**). Abdominocentesis was performed, followed by routine, biochemical, microbiological, and cytological examinations of the ascitic fluid. Acid-fast staining was negative for acid-fast bacilli, bacterial culture yielded no bacterial growth, and cytological examination showed no evidence of malignant cells.

**Figure 1:**
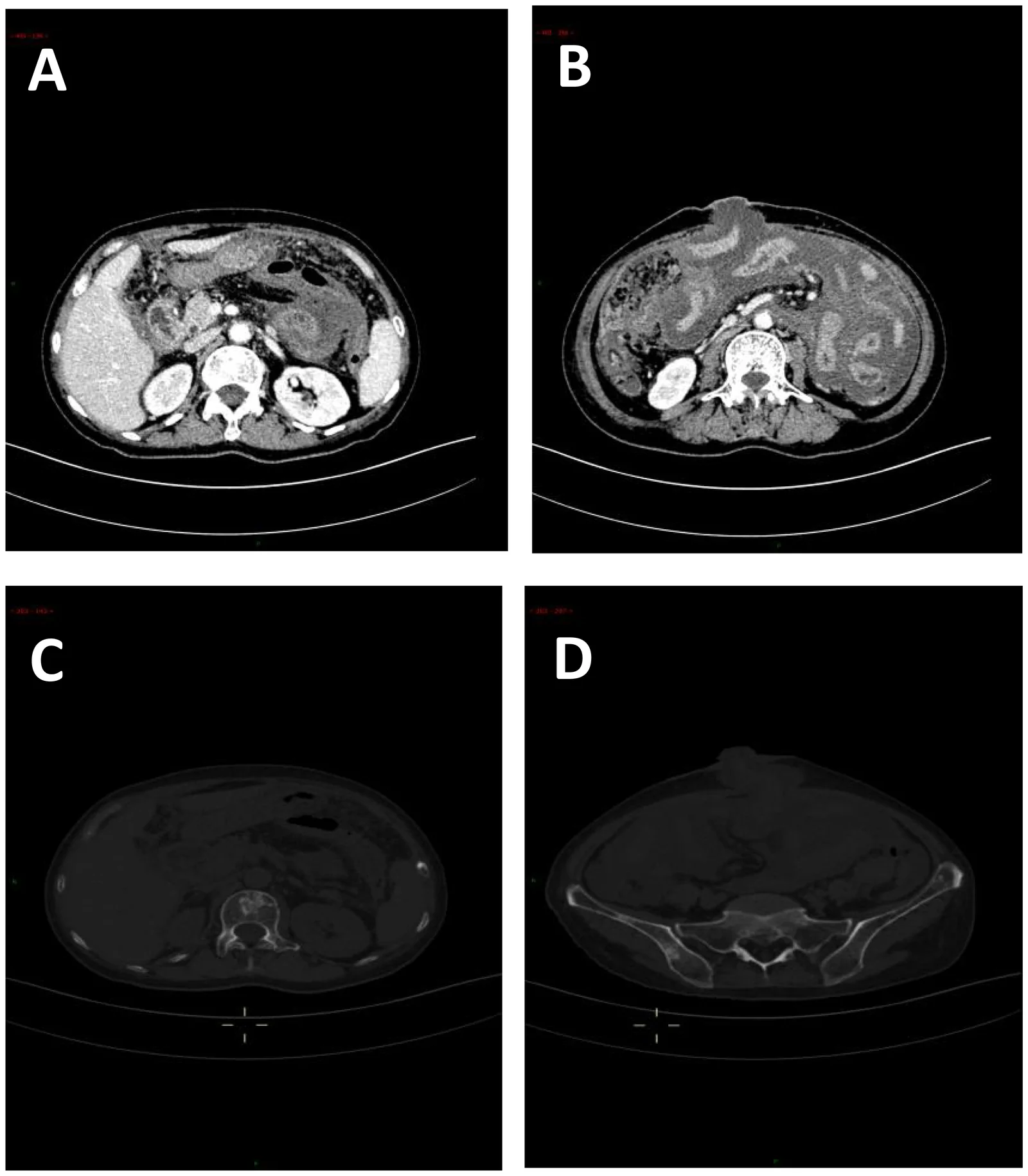
**(A, B)** Multiple areas of peritoneal thickening, partially exhibiting a "smudged" appearance, accompanied by ascites. **(C, D)** Patchy sclerotic lesions are observed in the L1 vertebral body and bilateral iliac bones.

Laboratory investigations indicated mild anemia (HGB: 98.8 g/L). Autoimmune serology showed positive ANA, anti-SSA (++), anti-Ro-52 (+) antibodies and elevated C4 levels. Additional immunological testing included complement C3, immunoglobulins, antistreptolysin O, rheumatoid factor (RF), and RF isotypes. Increased 24-hour urinary protein excretion (0.26 g/24 h) was also observed. Rheumatology consultation initially raised the possibility of UCTD based on the positive autoimmune serology. However, in the absence of characteristic clinical manifestations of a defined connective tissue disease, these findings were ultimately regarded as autoimmune serological abnormalities rather than evidence of an independent UCTD diagnosis. These abnormalities prompted rheumatologic evaluation and were considered in the decision to initiate immunomodulatory therapy, although their independent clinical significance remained uncertain.

To evaluate systemic involvement, the patient underwent a whole-body bone scan (SPECT), which revealed abnormally active bone metabolism in multiple sites, including the craniofacial bones, bilateral humeri, thoracic spine (T7-10), pelvis, and multiple areas of the bilateral femora and tibiae. Subsequently, a pathological review was performed on the specimens of the small intestine and omentum resected previously at the external facility. The results demonstrated extensive proliferation of foamy histiocytes in the submucosa and subserosa of the small intestine, accompanied by local mucosal ischemia, edema, vascular dilation, and fibroproliferation; Fibrous proliferation with chronic inflammatory cell infiltration was also observed within the omental adipose tissue. Immunohistochemical staining showed that the abnormal cells were positive for CD68 (+), *BRAF^V600E^* (+), and CyclinD1 (+);quantitative real-time PCR (qPCR) further confirmed the presence of the *BRAF^V600E^* mutation (**Figure 2**). Following multidisciplinary discussion involving gastroenterology, hematology, rheumatology, and pathology, the patient was definitively diagnosed with ECD.

**Figure 2:**
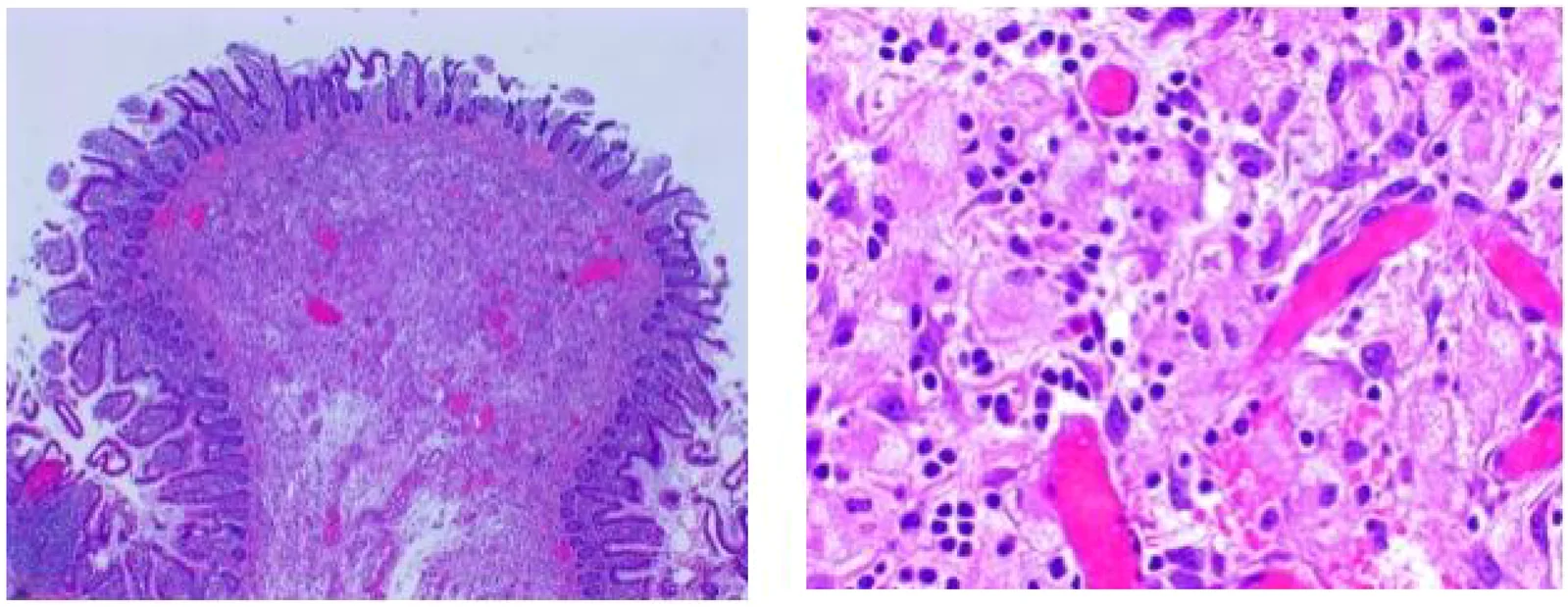
Extensive proliferation of foamy histiocytes is observed in the submucosa and subserosa of the small intestine, accompanied by ischemic changes in the overlying mucosa. The subserosa exhibits edema, vascular dilation and congestion, and focal fibroproliferation. Within the adipose tissue, fibrous proliferation is noted alongside chronic inflammatory cell infiltration and focal fibrinous exudation.

To comprehensively assess the extent of systemic involvement and exclude concurrent myeloid neoplasms, the patient subsequently underwent whole-body PET-CT and bone marrow aspiration. PET-CT revealed characteristic multisystemic infiltration of ECD (**Figure 3**): increased metabolic activity in multiple sclerotic lesions and diffuse hypermetabolism in the small intestine and peritoneum accompanied by massive ascites, confirming that the intestinal obstruction resulted from ECD infiltration. Furthermore, hypermetabolic lesions were observed around the aorta, pericardium, and right pleura. Comprehensive bone marrow evaluation showed increased bone trabeculae and focal myelofibrosis (MF grade 2) on biopsy, while flow cytometry and quantitative WT1 mRNA analysis successfully excluded concurrent CMML or MDS. Notably, flow cytometry revealed a decreased CD4/CD8 ratio (0.39), and the bone marrow smear showed toxic granulation and vacuolization in the myeloid lineage, suggesting an altered inflammatory and immune milieu (**Figure 4**).

**Figure 3:**
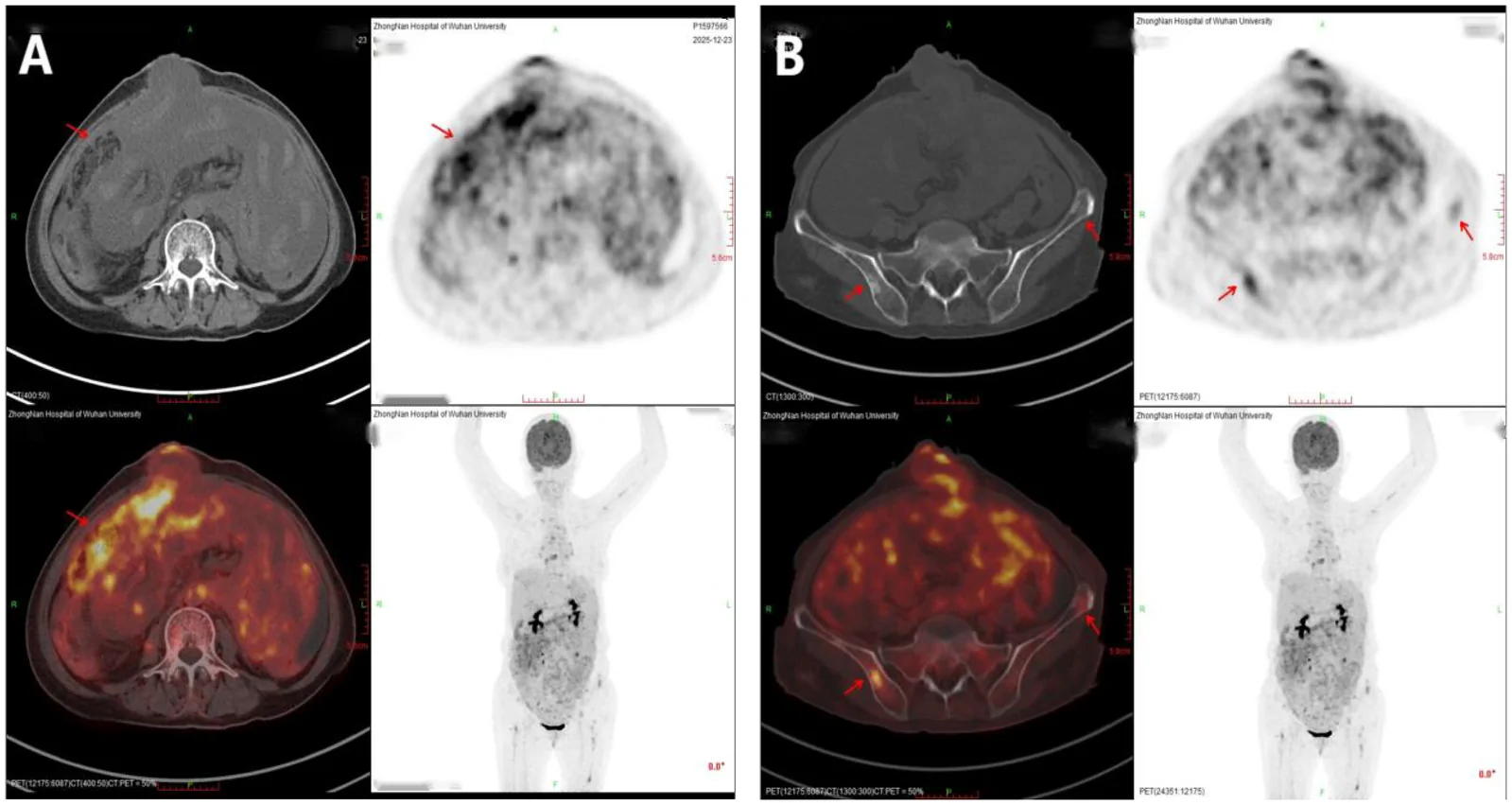
**(A)** PET-CT demonstrates multiple strandy thickenings involving the greater and lesser omenta, mesentery, bilateral paracolic gutters, and pelvic floor peritoneum, accompanied by diffusely increased radiotracer uptake in the small intestine (SUVmax 3.4 -7.4). **(B)** Furthermore, multiple patchy osteosclerotic lesions exhibiting intense radiotracer uptake (SUVmax 2.4 -6.3) are observed across the skeletal system, including the bilateral maxilla and mandible, sphenoid bone, left humerus, bilateral clavicles and scapulae, sternum, multiple vertebral bodies and posterior elements, pelvic bones, and bilateral femora.

**Figure 4:**
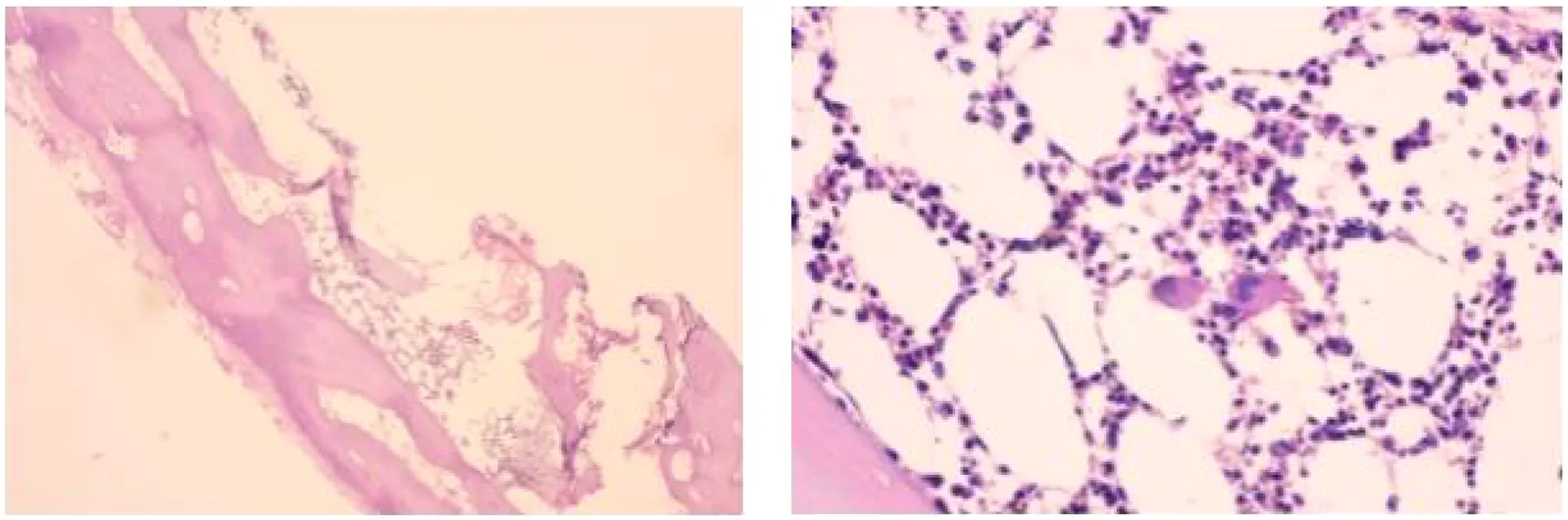
Bone marrow biopsy demonstrates increased and widened bone trabeculae. The overall bone marrow cellularity is normocellular, with active proliferation of the granulocytic, erythroid, and megakaryocytic lineages.

Following MDT discussion, the patient was ultimately diagnosed with multisystem *BRAF^V600E^*-mutated ECD presenting with abdominal cocoon syndrome. Based on this diagnosis, vemurafenib (480 mg twice daily) was initiated for ECD. In view of the associated immunological abnormalities, hydroxychloroquine sulfate (200 mg once daily) was also administered following rheumatologic evaluation. Three weeks after the initiation of treatment, the patient exhibited significant relief of abdominal pain and distension, with marked reduction in inflammatory markers (CRP decreased from 47.29 mg/L to 10.66 mg/L). Follow-up PET-CT was planned for 6 months after initiation of treatment; however, the patient had not undergone the examination at the time of manuscript preparation. The patient expressed profound relief following the targeted therapy, noting a significant improvement in her quality of life and expressing optimism toward her upcoming ostomy reversal surgery. The patient’s diagnostic and therapeutic course is summarized in **Figure 5**.

**Figure 5:**
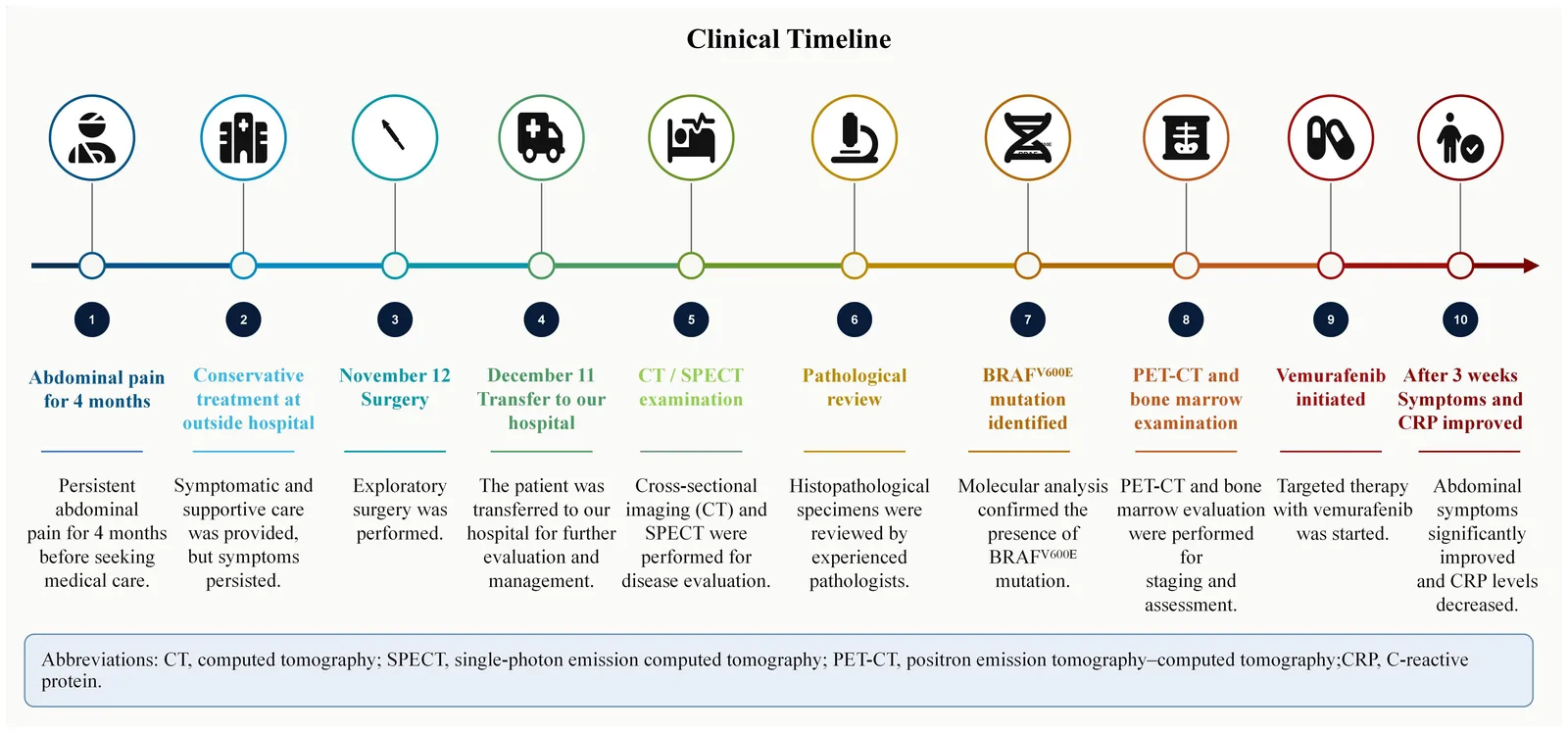
Clinical timeline of the diagnostic and therapeutic course.

## Discussion

ECD is a rare histiocytic neoplasm that predominantly affects adults. Despite presenting with classic manifestations such as osteosclerosis, hairy kidney and right atrial pseudotumor, the remarkable clinical heterogeneity of ECD often results in delayed diagnosis ranging from several months to several years (4, 8, 9). The present case is distinguished by its unusual gastrointestinal and peritoneal involvement presenting as abdominal cocoon syndrome. This presentation illustrates the diagnostic pitfalls when a systemic histiocytic neoplasm masquerades as a localized surgical emergency.

Gastrointestinal involvement in ECD is uncommon, and presenting as abdominal cocoon syndrome constitutes a highly deceptive clinical pitfall. Abdominal cocoon syndrome is characterized by the encasement of small bowel loops by a thick fibrotic membrane, which can be observed in idiopathic sclerosing encapsulating peritonitis, abdominal tuberculosis, peritoneal malignancy. However, in our instance, the macroscopic “cocoon” was actually a secondary manifestation of underlying histiocytic infiltration. Regarding its pathological mechanism, we hypothesize that histiocytic cells carrying the *BRAF^V600E^* mutation directly infiltrate the peritoneum and intestinal wall, promoting severe fibrosis; simultaneously, these cells recruit and activate Th1 cells, releasing substantial amounts of pro-inflammatory factors such as IL-6, TNF-α, and IL-1. This dysregulated pro-inflammatory cytokine signaling not only exacerbates inflammatory thickening and stenosis of the intestinal wall but also increases vascular permeability, leading to significant exudative ascites (10, 11).

Notably, the patient’s bone marrow flow cytometry revealed a decreased CD4/CD8 ratio (0.39), with toxic granulations observed in the bone marrow smear. In clinical immunology, such a ratio inversion typically indicates chronic immune exhaustion or autoimmune dysregulation (12). This provides potential evidence that the systemic cascade of inflammation driven by the *BRAF^V600E^* mutation clone may have altered the host’s immune microenvironment. Consequently, the presence of high-titer anti-SSA and Ro-52 autoantibodies in this case is likely a secondary immune dysregulation induced by the dysregulated cytokine triggered by underlying histiocytic proliferation.

Recent genomic studies indicate that approximately 10% of ECD patients concurrently present with classic overlapping myeloid neoplasms (such as CMML or MDS), which frequently share a common clonal hematopoietic origin (13). In this context, the comprehensive bone marrow evaluation conducted in this case successfully ruled out the possibility of concurrent myeloid tumors, which holds significant prognostic implications, as overlapping syndromes often exhibit more aggressive clinical courses and demonstrate poor response to pure BRAF inhibitor therapy (13).

Therapeutically, multiple modalities exist for ECD management, including targeted therapies, IFN-*α* treatment and cytotoxic chemotherapy (1, 14, 15). For patients with *BRAF^V600E^*-mutated ECD, BRAF inhibitors such as vemurafenib or dabrafenib are preferred therapeutic options, particularly in multisystem disease with end-organ involvement. For patients without *BRAF^V600E^* mutations, molecular profiling for other MAPK-ERK pathway alterations is recommended, and MEK inhibitors may provide effective targeted therapy (16). IFN-α/pegylated IFN-α and cytokine-directed therapies remain therapeutic options in ECD patients, while cytotoxic agents such as cladribine also remain important conventional first-line treatment options in selected patients (1). In the present case, identification of the *BRAF^V600E^* mutation provided a strong rationale for vemurafenib therapy, which was followed by rapid symptomatic improvement and a substantial decrease in CRP.

## Conclusion

In summary, we report a rare case of *BRAF^V600E^*-mutated multisystem ECD presenting with abdominal cocoon syndrome. Compared with previously documented cases, the uniqueness of this patient lies in the exceedingly rare intestinal involvement and the associated autoimmune serological abnormalities. This report not only underscores the pivotal value of integrating clinical features, imaging, pathology, and molecular testing in deciphering complex and challenging cases but also serves to broaden the differential diagnostic perspectives for gastroenterologists and gastrointestinal surgeons regarding the atypical intestinal manifestations of ECD.

## Data Availability

The original contributions presented in the study are included in the article/supplementary material. Further inquiries can be directed to the corresponding authors.
